# Recent Advances in Electrochemical Biosensors for Monitoring Animal Cell Function and Viability

**DOI:** 10.3390/bios12121162

**Published:** 2022-12-13

**Authors:** Kyeong-Mo Koo, Chang-Dae Kim, Fu Nan Ju, Huijung Kim, Cheol-Hwi Kim, Tae-Hyung Kim

**Affiliations:** School of Integrative Engineering, Chung-Ang University, Seoul 06974, Republic of Korea

**Keywords:** electrochemical biosensor, live-cell detection, anticancer assessment, stem cell differentiation monitoring

## Abstract

Redox reactions in live cells are generated by involving various redox biomolecules for maintaining cell viability and functions. These qualities have been exploited in the development of clinical monitoring, diagnostic approaches, and numerous types of biosensors. Particularly, electrochemical biosensor-based live-cell detection technologies, such as electric cell–substrate impedance (ECIS), field-effect transistors (FETs), and potentiometric-based biosensors, are used for the electrochemical-based sensing of extracellular changes, genetic alterations, and redox reactions. In addition to the electrochemical biosensors for live-cell detection, cancer and stem cells may be immobilized on an electrode surface and evaluated electrochemically. Various nanomaterials and cell-friendly ligands are used to enhance the sensitivity of electrochemical biosensors. Here, we discuss recent advances in the use of electrochemical sensors for determining cell viability and function, which are essential for the practical application of these sensors as tools for pharmaceutical analysis and toxicity testing. We believe that this review will motivate researchers to enhance their efforts devoted to accelerating the development of electrochemical biosensors for future applications in the pharmaceutical industry and stem cell therapeutics.

## 1. Introduction

Clinical monitoring and diagnostic methods that combine high sensitivity, specificity, and rapid detection with sample determination have been widely used [1,2,3,4]. Specifically, all human bodies and their processes are organized by cells, which are incredibly intricate biomachines [5,6,7]. Their fundamental processes (e.g., proliferation, division, differentiation, and death) should be studied to develop therapeutic approaches for a wide range of disorders and regenerate damaged tissues or organs [8,9,10,11]. With bioanalytical techniques, traditional biological principles have been integrated with digital instrumentation to create simple-to-operate portable devices [12,13,14,15]. These techniques guarantee that they can rapidly, accurately, and cost-effectively detect biologically related compounds through biorecognition and signal transduction [16,17,18]. A biosensor is an analytical tool that combines a biological component with a physicochemical detection transducer for detecting specific analytes, such as DNA and proteins [19,20,21,22]. Moreover, electrical and optical signals coupled with specialized biological responses involving isolated enzymes, tissues, organelles, or whole cells are used to detect chemical compounds or whole cells [23,24,25,26,27]. Several researchers have attempted to develop electrochemical-based biosensors that can rapidly detect whole cells and their dynamics in complex samples [28,29,30,31,32]. Since these developments, electrochemical biosensing has been extensively investigated for shifting medical paradigms (e.g., treatment and prevention) and diagnostic medicine because of live-cell monitoring [33,34,35,36].

Various electrochemical biosensor modes have been widely developed, and combinations of living cells and transducers have been utilized as cellular physiological signal transducers in various cells (e.g., highly proliferative cells) [37,38,39,40,41]. An electrochemical biosensor is a noteworthy integrated system that measures the quantitative and analytical profiling of a target via active interactions between biochemical compounds and an electrochemical transducer [42,43]. This entire process of electrochemical detection in living cells is considerably more effective and economical than conventional methods [44]. In addition, its performance for point-of-care (POC) label-free detection and miniaturization is remarkable [45,46]. Several studies have reported on the sensitivity and selectivity of electrochemical biosensing platforms; for example, (1) electric cell–substrate impedance sensing platforms (ECISs), (2) field-effect transistor sensors (FETs), and (3) potentiometric-based sensors have been enhanced [47,48,49]. The platform has been improved using various types of biosensors (e.g., ECIS-based, FET-based, and potentiometric-based biosensors) and biocompatible materials to address this issue. Consequently, the conductivity of the sensors improves, thereby increasing their sensitivity and selectivity [50,51,52,53]. Therefore, the limits of detection of the biosensors for live cells may be increased. A direct in situ detection approach is considered an analytical tool for living cells to underestimate the use of chemical agents (e.g., chemical dyes, radio-labeling, and fluorogenic probes) through electrochemical sensing in a non-destructive and label-free manner.

This method can be used to assess the viability of highly proliferative cells, such as cancer and stem cells, through their redox reactions; consequently, whole-cell detection has been improved [54,55,56,57]. With rapid screening and label-free detection, anticancer efficacy can be efficiently tested. Specifically, several studies have described the discovery and precise analyses of anticancer medication efficacy testing in 2D and 3D culture systems involving electrochemical methods [58,59,60]. Stem cell applications keep pace with the rapid advancement of medical technologies by using the genetic information of individuals to generate the same tissues used by the in vivo system and derived from patients. Hence, these cells exist in various types, such as pluripotent stem cells (PSCs), mesenchymal stem cells (MSCs), and embryonic stem cells (ESCs), which can differentiate into specific cells, such as osteoblasts, adipocytes, neurons, and cardiomyocytes [61]. During the differentiations, the successful differentiations and maturations should be electrochemically monitored for the application of these systems in regenerative medicine and stem cell-based therapeutics [62,63,64,65,66]. In addition, electrochemical biosensing can be used to monitor stem cell functions and differentiation without damaging cells or applying any labeling [57,67].

**Figure 1:**
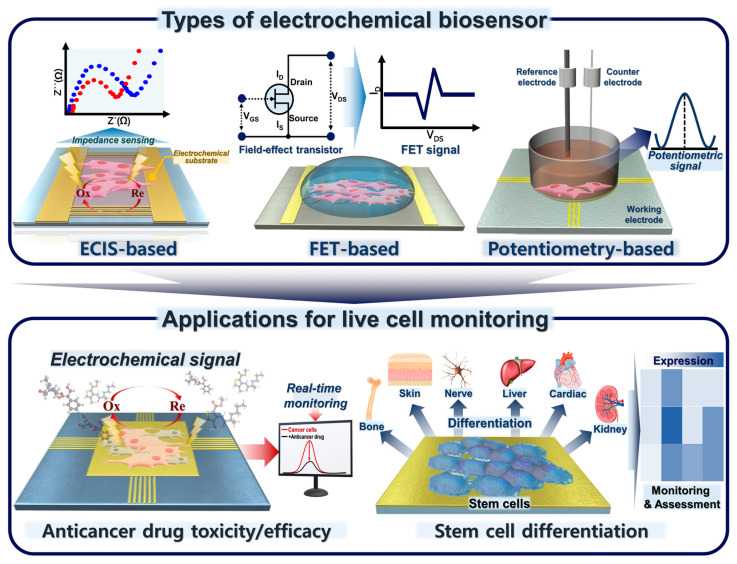
Schematic of various types of electrochemical biosensors for cancer/stem cell monitoring and their applications.

In this review, we discuss the development of electrochemical biosensors for monitoring living cells and their applications in cancer and stem cells (Figure 1). We also describe various types of electrochemical sensors integrated with a platform for cell detection. Electrochemical biosensors that can find living cells should be considered in future research on biomedicine and regenerative therapies.

## 2. Various Types of Electrochemical Biosensors for Live-Cell Detection

Electrochemical biosensors can detect and analyze the biological processes of living cells in a non-invasive and label-free manner because their measurement principles vary [68]. In this section, we describe various types of electrochemical biosensors, such as ECIS, FET, and potentiometric-based sensors, for the detection of live cells and biochemical substances from living cells. For instance, an electrochemical biosensor based on ECIS monitors the resistance between the cells and substrates [69]. ECIS-based electrochemical biosensors can be applicable to 3D-cultivated cells by simultaneously performing 2D and 3D cultures [70,71]. After 2D- and 3D-cultured HepG2 cells are exposed to various medications, the cell density and morphological alterations are evaluated using live–dead tests. Furthermore, FET-based electrochemical biosensors feature various quantifiable data and have fewer cell demands for the measurements [72,73]. Currently, electrochemical biosensors can monitor even at the single-cell level. As previously reported, the metabolic activity of human mammary cells is assessed using a PLL@G-FET electrochemical biosensor [74] that can detect the extracellular pH, which provides a more direct indication of the cellular environment; changes in the pH are then compared. Moreover, a potentiometric-based electrochemical biosensor can evaluate changes in extracellular environments by measuring the potential between a cell and the sensor in real time. H_2_O_2_, which is an essential component of apoptotic activity in several tissues and a participant in numerous intracellular processes, is assessed [75]. It is also used for the accurate measurement of H_2_O_2_ concentrations by increasing the conductive surface area [76,77]. This review summarizes the research progress on electrochemical biosensors and their applications by using various detection methods, including amperometric, differential pulse voltammetry (DPV), and linear sweep voltammetry (LSV; Table 1).

### 2.1. ECIS

ECIS-based biosensors are electrochemical tools used to examine cell adhesion, proliferation, growth, and viability in real time by measuring the impedance between a cell and a substrate. One way to determine this parameter is by measuring the resistance between the cell and the substrate. Therefore, the quality of a cell culture and cell-based assays in vitro may be improved by increasing the cell–substrate interactions. Traditional cell-based assays, such as WST-1, MTT, and BrdU, are not only indirect but also arduous, time-consuming, and intrusive [92]. Impedance sensing can be used to investigate the kinetic elements of this complicated process. Cell–substrate adhesion with various ECMs, such as laminins, fibronectin, and collagen, is one of the cell–ECM interactions that may be studied using ECIS. ECIS can also be used to investigate other cell–ECM interactions. During ECIS, a sinusoidal voltage with a frequency of 10 kHz is applied to interdigitated electrodes (IDEs), thereby forming an ion current between the IDEs. At low frequencies, cells are regarded as non-conductors because of their properties. When the cells connect to and begin to develop on the IDEs, the ion current is blocked, and the impedance of the IDEs increases. As the cells die, they become separated from the IDEs, resulting in a decrease in the impedance. Because the cells cannot directly adhere to or detach from the typical IDEs, monitoring 3D-grown cells encased in Matrigels is difficult when conventional IDEs are used because the cells cannot attach to or detach from the IDEs. Consequently, conventional techniques used for monitoring 2D-grown cells are unsuitable for monitoring 3D-grown cells. Therefore, high-throughput, real-time, non-invasive, and label-free methods of monitoring 3D-grown cells should be developed.

**Figure 2:**
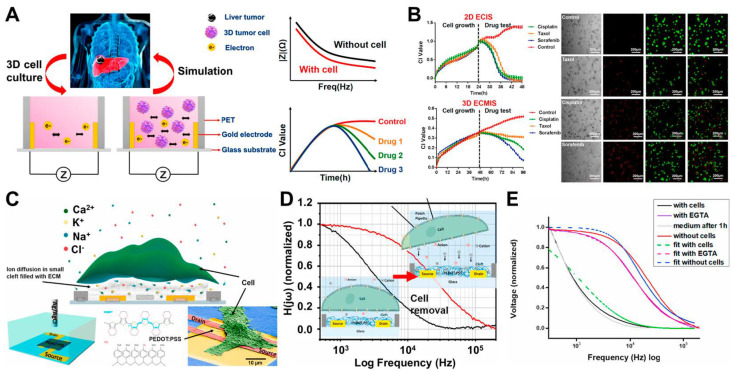
(**A**) Principle of the 3D ECMIS system for live-cell monitoring. (**B**) Cell-growth curves of 2D/3D-cultured HepG2 cells exposed to various anticancer drugs (cisplatin, taxol, and sorafenib) on a 2D/3D ECIS. Changes in the cell density and morphological characteristics of the 3D-cultured HepG2 cells analyzed by live–dead staining at 96 h. (**C**) Schematic of PEDOT:PSS OECT devices and a colored scanning electron microscopy (SEM) image of a fixated HEK 293 cell. (**D**) Detaching the cell from the OECT gate increases the low-pass frequency of the device’s transfer function from 2 kHz to greater than 10 kHz. (**E**) Strong attenuation of the spectrum with MDCK cells adhered to the OECT (black), added with EGTA (purple), removed from the surface (red), adherent cells (green), spectrum with opened gap junctions (pink), and fitted cells (blue). Reprinted with permission from [70]. Copyright 2019, Elsevier; reprinted with permission from [75]. Copyright 2021, Elsevier.

The evaluation of the efficacy and toxicity of drug candidates in preclinical settings plays a key role in drug discovery and development. Traditional planar cell culture is a typical method used for preclinical drug testing; however, because of the simple 2D extracellular milieu, accurately predicting the efficacy and toxicity of a drug is difficult. A 3D cell culture system can better simulate the complex extracellular milieu that exists when the cells live in 3D tissues and organs in vivo than a planar cell culture system. Thus, Pan et al. developed 3D electric/cell Matrigel–substrate impedance sensing (3D ECMIS) for the real-time and non-invasive monitoring of 3D cell viability and drug susceptibility (Figure 2A) [70]. An alternating current (AC) mode is utilized by the 3D ECMIS to evaluate the impedance of a 3D cell/Matrigel construct. The 3D ECMIS is composed of a pair of vertical gold electrodes. During this time, an 8-channel 3D ECMIS detection system is built so that changes in the impedance of the 3D cell/Matrigel construct can be recorded. As predicted, the monitoring of cellular activities involves measuring the changes in impedance between the vertical electrodes. The size and working frequency of the vertical electrodes are adjusted to increase the sensitivity of the 3D ECMIS. In addition, a comparison is carried out between the 3D ECMIS and conventional optical detectors. The impedance readings are normalized with the cell index (CI), which is the ratio of cell growth-induced Z-value changes to the baseline impedance (Z_0_), by using the following equation: CI = ΔZ/Z_0_. Furthermore, the 3D ECMIS can be used to assess the anticancer drug efficacies in 3D-cultured tumor cells. The conventional 2D culture of cells does not consider the natural 3D microenvironment of the cells in vivo. Three anticancer drugs with varying degrees of efficacy are chosen to test the 2D- and 3D-cultured models of the HepG2 cell line and to determine whether the 3D ECMIS can provide more reliable and accurate data for drug screening. These drugs include taxol, which is used to treat ovarian and breast cancers, and cisplatin, which is utilized to treat broad-spectrum cancers. Sorafenib is described in Figure 2B. These medications have flawless efficacy when they are applied to 2D-cultured HepG2 cells; nevertheless, the efficacies of all three treatments applied to patients with liver cancer are distinct. However, differentiating the efficacies of these three medications by using the 2D ECIS is challenging. Meanwhile, the 3D ECMIS may reflect the various reactions of 3D-grown HepG2 cells to these medications (Figure 2B). Amongst these drugs, sorafenib has the most favorable effects on the 3D HepG2 cells. This conclusion is further supported by the findings presented in fluorescent images. Experimental results have demonstrated that the performance of the 3D ECMIS is great for anticancer drug screening in vitro. With this 3D cell-based biosensor, the growth and viability of 3D cells can be easily tracked, and drug screening data can be more accurately obtained. This 3D cell-based biosensor can also be used to improve the accuracy of cell-based cancer drug screening. Thus, the 3D ECMIS will be a high-throughput, non-invasive, real-time platform for 3D cell monitoring and screening of anticancer drugs.

Various biomedical processes, including wound healing, cancer formation, and immune system recognition, rely on cellular adhesion and cell–cell interactions. ECIS is a well-established method in this research field [93]. It is used to assess the adhesion strength of cells on technological substrates or qualify the barrier function of confluent tissues. Both functions are necessary to determine how efficiently cells adhere to their respective surfaces. Due to the increase in the impedance of noble metal microelectrodes as their size decreases, electrode downsizing to a single-cell diameter (about 10–20 μm) is a core challenge in ECIS. Thus, Hempel et al. [75] proposed the adhesion of cells to organic electrochemical transistors (OECTs), which are fabricated by using poly(3,4-ethylenedioxythiophene):poly(styrene sulfonic acid) (PEDOT:PSS). As shown in Figure 2C, this proposed platform demonstrates the overarching idea that underpins the various tests outlined in this study. In OECTs, changes in the conductivity of the polymer are caused by the diffusion and intercalation of cations, such as sodium, potassium, and calcium, into the polymer layer; such changes can also be attributed to their binding to the pending sulfonate anions of the PSS. The cations are forced towards the polymer layer, which serves as a gating mechanism for the devices, whenever a positive voltage is given to the reference electrode. This electrochemical gating technique can also be employed for the detection of cellular adhesion, although it differs significantly from the field-effect gating mechanism of silicon-based platforms. The adherence of the cells to the OECTs creates a small fissure between the connected portion of the cell membrane and the device surface, which is typically between 50 and 100 nm wide and packed with proteins and ECM components. In this area of thin adhesion, the free movement of ions towards and away from the polymer gate of the OECT is modified. Then, the bandwidth of the OECTs generated is optimized by introducing a novel fabrication technique. As the final step of the fabrication, a spin-coating technique is paired with a “light” lift-off by using acetone to produce PEDOT:PSS layers with a thickness between 10 and 20 nm. Figure 2D illustrates the mechanical removal of a single cell from a 20 × 20 μm OECT gate while the remaining cells on the contact lines remain unaffected. Ethylene glycol-bis(2-aminoethylether)-N,N,N′,N′-tetraacetic acid (EGTA) is a calcium-chelating chemical that can be used to reversibly control adherent MDCK cells. The impedance spectrum significantly decreases in cell culture media. The low-pass properties of the spectrum increase after EGTA is introduced. The spectral form reverts to its original state when the EGTA is removed, and the measurements are repeated in cell media. This behavior can be explained by the interaction between R_J_ and R_Seal_ in the equivalent circuit. As predicted, the low-pass characteristics significantly increase when the cells are destroyed by trypsin (Figure 2E). Thus, the OECT is modelled for single-cell-level cellular impedance sensing. OECTs are useful for drug screening and cytotoxicity testing because of their low cost and ease of use.

### 2.2. FET-Based Electrochemical Biosensors

Among the different forms of biochemical sensors, the combination of biological elements and an FET is the most intriguing. In 1970, an FET without a gate metallization was designated as an ion-sensitive FET (ISFET) for detecting ion concentrations [94,95]. This modified FET was classified as an oxide semiconductor field-effect transistor (OSFET). In the metal oxide semiconductor (MOS) transistor configuration that makes up the OSFET, the gate metal has been removed. Thus, the OSFET can be directly utilized as an active probe in the extracellular fluid. The FET sensor has evolved into a biosensor based on individual cells. In addition to the ionic effluxes around a neuron, metabolic parameters, such as oxygen consumption, are available for detection. This expands the range of parameters that can be measured. In the last 10 years, considerable advancements have been made in semiconductor technology. As a result of these advancements, FET-based devices have lower costs and more reliable performances. In medicine and biotechnology, cell-based FET sensors are utilized in various applications. In this part of the review, we focus on the monitoring of the cell’s microenvironment and the detection of the cell’s electrophysiological activity.

**Figure 3:**
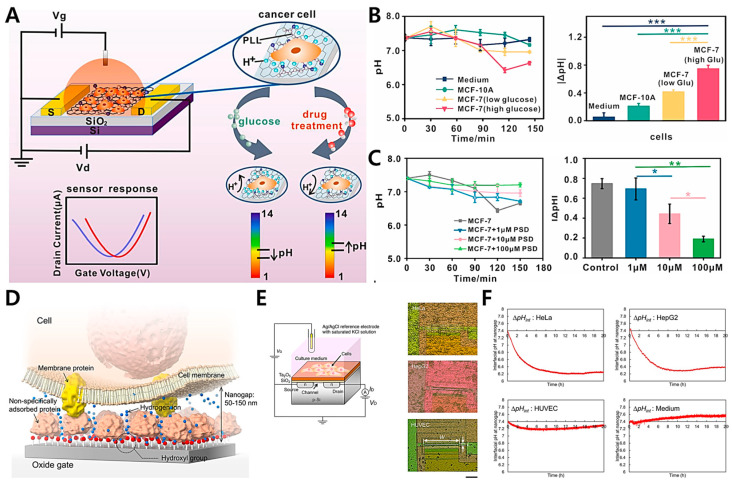
(**A**) A PLL@G-FET electrochemical biosensor that measures extracellular pH. (**B**) Monitoring of cancer cell metabolism. Comparing the extracellular acidification and pH of MCF-10A cells with those of MCF-7 cells. (**C**) Identification of the extracellular acidification of MCF-7 cells without drug treatment and with different drug concentrations (left panel), and pH changes with the intervention of various drug concentrations within 150 min (right panel). (**D**) A diagrammatic representation of the cell/gate nanogap interface. (**E**) Cell-coupled gate ISFET (left panel); HeLa, HepG2, and HUVEC cells were grown on the Ta_2_O_5_ gate (right panel). (**F**) Changes in interfacial pH at the cell/gate nanogap are measured for each cell by using a cell-coupled gate ISFET biosensor. Reprinted with permission from [74]. Copyright 2022, Elsevier. Reprinted with permission from [73]. Copyright 2018, Royal Society of Chemistry.

The detection of extracellular microenvironments is crucial for the diagnosis of cancer cells. Using the suggested platform, Xiao et al. [74] proposed a graphene field-effect transistor modified with poly-l-lysine (PLL@G-FET) for the real-time monitoring of changes in an extracellular acidic environment by using cultured cancer cells (Figure 3A) [74]. This PLL@G-FET biosensor is highly sensitive to changes in hydrogen ion concentrations; consequently, the pH of cancer cells can be monitored in situ. Specifically, the glucose content in cell culture media is increased to examine the effect of glycolysis on the extracellular acid environment (Figure 3B). Therefore, the extracellular acidification of MCF-7 cells cultivated in a medium containing a high glucose concentration decreases dramatically, and the pH changes by 0.75. Figure 3C shows the graphs of the pH changes of the MCF-7 cells treated with various drug concentrations for 150 min. Without medication, the pH of the cancer cells changes by 0.75, while the concentrations of pulsatilla saponin D (PSD) alter the pH by 0.69 and 0.44. The MCF-7 cells are only inhibited by pH 0.33 when they are exposed to 100 M PSD at high concentrations. These results demonstrate that cancer cell metabolism is dependent on aerobic glycolysis. In addition, this FET sensor is utilized to monitor the changes in extracellular pH in response to pharmacological intervention; therefore, PSD may suppress the glycolysis-dependent metabolism of cancer cells.

The Warburg effect is well known because cancer cells generate adequate adenosine triphosphate (ATP) through glycolysis rather than oxidative phosphorylation (OxPhos) [96]. Specifically, aerobic or anaerobic cellular respiration involves a series of metabolic reactions to create ATP from the uptake of a resource (e.g., glucose); thereafter, the live cells release waste products, such as carbon dioxide or lactate [73,97]. Cancer cells exhibit extracellular acidification; hence, their microenvironmental pH differs from that of normal cells. Traditional approaches (such as fluorescent probes and optical devices) have been developed to image and measure the pH around tumors. However, most of these procedures are costly and time-consuming. Sakata et al. proposed an ion-sensitive FET (ISFET) for monitoring cellular respiration (Figure 3D) [73]. This platform enables the label-free, non-invasive, and real-time monitoring of pH behaviors in the immediate vicinity of cancer cells. The gate insulator, which is used as an electrode, usually consists of an oxide with hydroxyl groups at the surface in a solution, and the ISFET platform is sensitive to changes in the concentration of positively charged hydrogen ions based on the equilibrium reaction. For electrical measurements with the ISFET platform, changes in the V_G_ in V_G_-I_D_ electrical characteristics are assessed as a threshold voltage (V_T_) shift, which is examined with a constant drain voltage (V_D_) of 2 V and a constant I_D_ of 700 μA. A salt bridge connects an Ag/AgCl reference electrode with a KCl solution to the measuring solution (Figure 3E). With the ISFET (Figure 3F), the cellular respiration can be directly monitored as ΔpH_int_ at the cell/gate nanogap interface. The ΔpH_int_ of cancer cells (HeLa and HepG2 cells) changes by a factor between 5 and 6, which is greater than that of normal cells (HUVECs). As demonstrated by the above data, the ΔpH_int_ of cancer cells is greater than that of normal cells. Therefore, the ionic behaviors at the cell/gate nanogap interface should be prioritized for the real-time monitoring of cellular respiration by using the ISFET sensor. Thus, the nanogap interface is considered to be the closed nanospace between the cell and the gate, in which the released ions and biomolecules are concentrated; as a result, the H^+^ concentration increases during cellular respiration. Accordingly, allowing for the development of a cell-coupled gate ISFET sensor is ideal for the real-time and label-free monitoring of cell metabolism in various cell types. The electrical approach for measuring the pH of the microenvironment around cancer cells based on extracellular acidosis is particularly attractive.

### 2.3. Potentiometric-Based Electrochemical Biosensors

**Figure 4:**
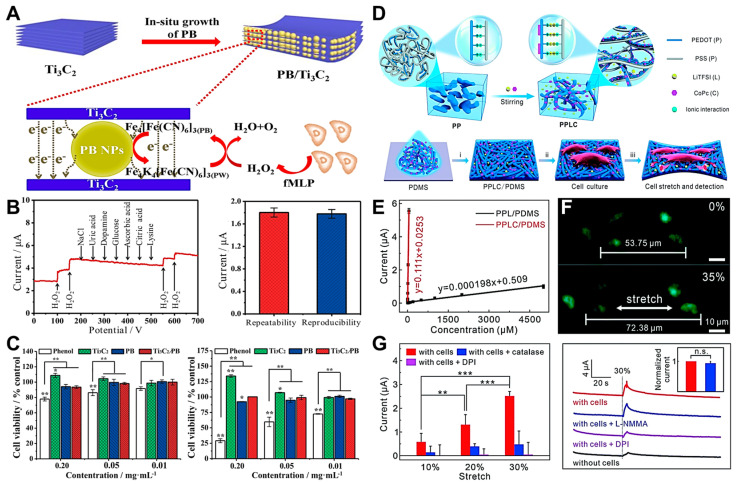
(**A**) Schematic of a PB/Ti_3_C_2_/GCE biosensor for detecting H_2_O_2_ from live HeLa cells. (**B**) Amperometric I–T response of the PB/Ti_3_C_2_/GCE biosensor with the consecutive treatment of 100 μM of H_2_O_2_ (left panel). Current response of the amperometric detection for 0.2 mM of H_2_O_2_ (right panel). (**C**) Cell viability of L929 cultured with different concentrations of nanomaterials for 5 h (left panel) and 48 h (right panel; * *p* < 0.05 and ** *p* < 0.01). (**D**) Schematic of the PEDOT:PSS platform functionalized with LiTFSI and CoPc for monitoring cells by detecting H_2_O_2_ from live cells. (**E**) Calibration graph of the PPL/PDMS and PPLC/PDMS electrodes at various H_2_O_2_ concentrations. (**F**) Representative fluorescent images of 16HBECs cultured on a PPLC/PDMS film stained with Calcein-AM (green) and PI (red) before and after they are stretched. (**G**) Amperometric responses detected from 16HBECs to various stretch stimuli (left panel), and 30% strain with various treatments at a potential of +0.55 V (vs. Ag/AgCl; right panel). Reprinted with permission from [77]. Copyright 2020, Elsevier; reprinted with permission from [76]. Copyright 2021, Royal Society of Chemistry.

Electrochemical biosensors with a potentiometric basis have been developed for the accurate detection of target substances in live cells [98,99]. They are utilized to determine the electrochemical potentials of metallic structures under specific conditions. Among the target substances of living cells, H_2_O_2_ is not only an essential by-product of various metabolic events but also an essential starting material or intermediate product in numerous different chemical and biological reactions. In addition, the H_2_O_2_ concentration in cells may be utilized as a reliable indicator of physiological activities, and aberrant amounts of H_2_O_2_ are the primary causes of several disorders. Therefore, the H_2_O_2_ content in live cells should be monitored accurately and efficiently to offer regular diagnostic criteria for targeting diseases. Dang et al. proposed a potentiometric-based electrochemical sensing system to detect H_2_O_2_ levels in living cells in real time (Figure 4A) [77]. Different potentials (−0.2, 0.1, 0.0, and 0.1 V) are individually applied in current analyses when 100 μM of H_2_O_2_ is continuously applied to PB/Ti_3_C_2_/GCE electrodes to achieve the desired sensitivity of the H_2_O_2_ sensors. A hierarchically hybrid nanostructure is constructed by growing Prussian blue (PB) nanoparticles (NPs) between Ti_3_C_2_ nanosheets in situ to combine the metal-like conductivity of Ti_3_C_2_ with the high catalytic activity of H_2_O_2_ from PB and Ti_3_C_2_. A non-enzymatic H_2_O_2_ biosensor is designed to sensitively and selectively monitor the H_2_O_2_ released from living HeLa cells. The most significant characteristic of the sensing interface is its capacity to differentiate specific analytes from other potential interfering chemicals. Hence, the selectivity of PB/Ti_3_C_2_/GCE towards H_2_O_2_ has been investigated. After an electrode is triggered, an amperometric I–T curve (Figure 4B) is drawn to examine the effects of numerous common interfering chemicals on the current response of H_2_O_2_ reduction at a constant potential of 0.0 V. In a 0.1 M PBS buffer solution, 10-fold co-interfering chemicals (e.g., NaCl, uric acid, dopamine, glucose, ascorbic acid, citric acid, and lysine) are consecutively administered. When 100 μM of H_2_O_2_ is added, rapid and noticeable current responses are observed. Regarding the continuous addition of biologically active compounds, no significant reaction current is observed. In addition, the reduction current response of H_2_O_2_ is slightly perturbed, even in the presence of the interfering compounds, and a distinct current response occurs as H_2_O_2_ is further added, indicating that PB/Ti_3_C_2_/GCE possess excellent selectivity to H_2_O_2_. For a biocompatibility assessment, healthy fibroblast cells are co-cultured with varied doses of PB, Ti_3_C_2_, and PB/Ti_3_C_2_ solutions for 5 and 48 h. In Figure 4C, an MTT assay is performed to assess the cytotoxicity of each group. After 5 h of culture, the phenol group is strongly dependent on concentration, and the cell survival rate decreases dramatically as the phenol concentration increases. This result becomes increasingly apparent when the culture time is 48 h. As the co-culture concentration increases from 0.01 mg/mL to 0.20 mg/mL for PB, Ti_3_C_2_, and PB/Ti_3_C_2_, the cell viability is marginally affected. Surprisingly, the survival of L929 cells grown in Ti_3_C_2_ slightly increases as the Ti_3_C_2_ concentration increases. This trend becomes more obvious when the cultivation time increases to 48 h, proving that Ti_3_C_2_ helps cells grow.

Numerous studies have been conducted to measure and monitor metabolic intermediates, such as glucose, lactate, and H_2_O_2_, from live cells [100,101]. Cells in the body are continually exposed to mechanical stresses, which may be detected and converted into biochemical signals. Because of the crucial role of mechanical forces in cell activity and mechanical signaling, cell mechanotransduction has been widely investigated. Stretchable electrochemical biosensors have been substantially improved for investigating dynamic mechanotransductions; furthermore, evoked chemicals, such as H_2_O_2_ and nitric oxide (NO), from endothelial cells and inflated intestines have been effectively monitored. Yan et al. developed a live-cell detection platform and discussed force-activated signaling in mechanotransduction by using a potentiometric-based electrochemical biosensor (Figure 4D) [76]. Specifically, a strategy for fabricating PEDOT-based stretchable electrochemical biosensors is developed. A stretchable PPLC/PDMS biosensor is utilized to detect mechanically induced H_2_O_2_ released from human bronchial epithelial cells and load strains in situ. The amperometric assessments of a series of H_2_O_2_ solutions with increases in their concentrations reveal that CoPc molecules are superior to H_2_O_2_ electro-oxidation (Figure 4E). The PPLC/PDMS electrode is sensitive to 200 nM of H_2_O_2_ and has a strong linear relationship with H_2_O_2_ across a wide concentration range (from 200 nM to 50 mM). Then, the electrode containing 16HBECs is subjected to a tensile strain of approximately 35% to fully cover the physiological strain ranges (5–15%). The fluorescence staining of Calcein-AM and PI demonstrates that the cells adhere securely to the substrate and remain alive throughout the stretching (Figure 4F). Additionally, the stretch-evoked release of H_2_O_2_ from the live cells increases the magnitude of stretching; conversely, the suppression of the NADPH oxidase activity by diphenyleneiodonium chloride (DPI) results in a negligible signal. The current response recorded from patients treated with L-NMMA, which is an inhibitor of NO synthase, shows no significant decrease, although it is considerably reduced in the absence of DPI therapy (Figure 4G). In conclusion, this platform is expected to provide a wide range of options for polymer-based flexible and wearable sensors that can be used in biological engineering and medical applications.

## 3. Application for Live-Cell Monitoring Based on Electrochemical Biosensing

Larger-unit cells should be identified for their use in disease modeling and the development of therapeutic and preventative measures; furthermore, electrochemical approaches are likewise useful [34]. However, the development of biosensors that can detect biological changes is more challenging than that of biomolecules since the structure and content of a delicate cellular microenvironment shift in response to changes in the culture conditions [102], including temperature, pH, and nutrient availability. Although certain investigations render living samples irretrievable, conventional techniques such as immunostaining, polymerase chain reaction (PCR), and flow cytometry analysis (FACS) are widely employed to define cells and tissues [103,104]. Biosensors based on electrochemical methods have been extensively studied for their potential applications in drug screening and regenerative medicine. Electrochemical biosensors are developed to detect the anticancer effects of diverse medications, evaluate novel drug candidates, and create potent anticancer drug candidates [105]. In addition, electrochemical biosensors have only become available for sensing living stem cells. The pluripotency and differentiation of stem cells should be monitored rapidly and non-destructively [106]. The therapeutic potential of stem cells can be evaluated by using techniques such as electrochemical biosensing in regenerative medicine to measure osteogenesis and neurogenesis. The stem cell culture must be optimized for enhanced sensitivity, selectivity, and ease of manipulation to facilitate this more advanced research effort. Therefore, the development of this new method to improve electrochemically based technologies will be essential for the commercialization of stem cell-based biosensors. This review summarizes the research progress on the application of electrochemical biosensors in rapidly dividing cell types (e.g., cancer and stem cells) by using various detection methods, such as EIS, amperometric, cyclic voltammetry (CV), scanning electrochemical microscopy (SECM), and square wave voltammetry (SWV; Table 2 and Table 3).

### 3.1. Cancer Cell-Based Sensing and Assessment of Anticancer Drug Candidates

Pharmacokinetic studies should be performed to accurately analyze the effects of drugs on target cells and determine the best drug candidates [119]. Conventional drug screening methods and tools such as live–dead staining, PCR, flow cytometry, and colorimetric assays are widely used to assess the toxicity and efficacy of drug candidates. However, these techniques are incapable of real-time monitoring and rapid assessment without compromising cell viability. To counteract this limitation, researchers have used electrochemical methods (e.g., EIS and DPV methods) for the non-invasive, real-time, and high-throughput pharmacokinetic assessment of cell viability, proliferation, and cytotoxicity [120].

**Figure 5:**
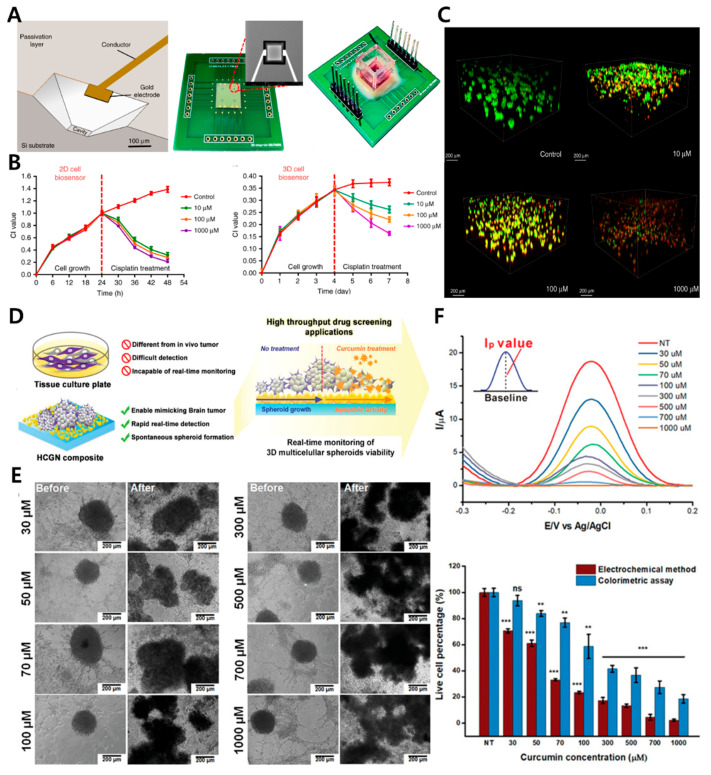
(**A**) Construction of a 3D microgroove impedance sensor (MGIS) and a real image of the complete sensor with 3D cell culturing. (**B**) Real-time monitoring of the anticancer drug effects on 2D/3D lung cancer models. (**C**) Live–dead cell analyses of exposure to cisplatin at certain concentrations on day 7. (**D**) Schematic of a highly conductive gold nanostructure (HCGN) biosensor for the viability of 3D multicellular spheroids. (**E**) Microscopy images of 3D-cultured spheroids before/after treatment with curcumin at specific concentrations. (**F**) Electrochemical assessments of the toxicity of curcumin in 3D-cultured spheroids. Reprinted with permission from [121]. Copyright 2020, Springer Nature; reprinted with permission from [58]. Copyright 2020, Wiley Online Library.

Using 3D lung cancer models, Pan et al. suggested a multidimensional microgroove impedance sensor (MGIS) for the drug screening of chemotherapeutic candidates (Figure 5A) [121]. They described the 3D ECIS approach for 3D cell monitoring and the accurate evaluation of drug efficacy for individualized therapy. They also used a typical micro-electromechanical system (MEMS) to manufacture an MGIS chip. According to Morgan’s theory, an equivalent circuit model comprising a double-layer capacitance between an electrolyte and an electrode (C_DL_), the resistance of the Matrigel (R_m_), the capacitance of the cell membrane (C_BLM_), and the resistance of the cell (R_c_) is designed for impedance analyses in 3D cells. The apoptosis in the 3D lung cancer model reduces the number of gap junctions, leading to an increase in the 3D cell/Matrigel construct’s impedance. On the basis of this approach, the capability of the 3D ECIS to monitor 3D apoptosis in cells is further investigated. A stable 3D model of lung cancer is also constructed using the previously adjusted cell density for apoptosis in anticancer drug testing. Cisplatin, a widely used chemotherapeutic agent for lung cancer, can bind to DNA and produce cross-linking, thereby damaging DNA function and limiting cell division [122]. Furthermore, 2D-A549 and 3D-A549 cells in their growth plateau are treated with 10, 100, and 1000 μM of cisplatin. In the 2D cells, all of the cisplatin doses are effective. However, the 3D ECIS can differentiate the effects of the three cisplatin doses on the vitality of the 3D lung cancer models. The highest cisplatin concentration has the greatest effect on the CI of the 3D cells. In the lung cancer models, the maximal drug efficacy of all tested concentrations of cisplatin is evaluated; extremely significant differences are identified between the 2D ECIS (10 μM: 77.11% ± 0.58%; 100 μM: 80.67% ± 0.88%; 1000 μM: 84.69% ± 0.89%) and the 3D ECIS (10 μM: 30.44% ± 0.87%; 100 μM: 44.38% ± 2.03%; 1000 μM: 57.73% ± 0.89%; Figure 5B) because the 2D cells lack an extracellular matrix and intercellular connections occur in the 3D in vivo environment. Fragile monolayer cells can be treated directly with anticancer medicines. As 3D cells have a natural form in spheroid/aggregate structures, medications may fail to fully penetrate the spheroids and, therefore, cannot reach the cells close to the core. Consequently, 2D cells are frequently susceptible to low drug concentrations, and the capacity of 2D cells to discriminate the efficiency of anticancer medicines at varied doses is lower than that of 3D cells. In addition, in vitro 2D cell results can occasionally give false-positive findings that do not accurately represent the outcomes of 3D cell assays and in vivo trials. Fluorescence confocal tests have been used to validate the accuracy of the MGIS chip’s cell apoptosis measurements. Similar to the findings of the 3D ECIS, Figure 5C shows that the different cisplatin doses have varying effects on the apoptosis of the 3D-A549 cells. Previous results have determined that the 3D cellular responses to therapeutic treatments are more similar to in vivo responses than the 2D cellular responses, and 3D cells are more resistant to anticancer medicines than 2D cells. For instance, after paclitaxel therapy, ovarian cancer cells survive between 40 and 60% less frequently in 3D cells; however, the same treatment results in an 80% decrease in cell survival in 2D cell monolayers. Tailored tumor microenvironments based on 3D cell culture systems have been engineered to predict the clinical responses in an independent validation group of 55 patients, demonstrating a high degree of sensitivity. This proposed technique shows that the 3D ECIS can be an efficient method for predicting in vivo anticancer effects. The clinically observed effects of single-agent and combination therapies for non-small-cell lung cancer closely resemble the findings of this study. Therefore, organoid technology should be used to imitate in vivo solid tumors and should be related to the MGIS chip to develop organoid-based biosensors for pharmacological screening so that precise medicine can be achieved more effectively.

Suhito et al. proposed a bio-multifunctional platform for 3D multicellular cancer spheroid production and a real-time assessment of anticancer medication (Figure 5D) [58]. HAuCl_4_ is electrodeposited onto an indium tin oxide (ITO) glass electrode. Highly conductive gold nanostructures (HCGNs) are the basis of this platform, which promotes the spontaneous development of spheroids and allows the electrochemical detection of their viability. Gold nanostructures facilitate automatic spheroid development because their rough surfaces reduce cell adherence. In addition, gold nanoparticles are attractive candidates for electrochemical detection because of their high conductivity, long-term stability, and excellent biocompatibility. SH-SY5Y and U87-MG cells are co-cultured to generate spheroids by using DPV at varying cell-to-cell ratios. Furthermore, this co-culture spheroid system on a multifunctional platform is used for anticancer drug screening. Optical microscopy shows that co-cultured spheroids are more resistant to low curcumin concentrations (30–50 μM). At 30 and 50 μM concentrations of curcumin (Figure 5E), the DPV signals decrease to about 29.4% and 38.9%, respectively, while the CCK-8 assay reveals that viability decreases by 6.3% and 16.2% at the same dosages. At 70 μM of curcumin, the spheroid viability has a statistically significant difference, which is that DPV and CCK-8 reveal 66.7% and 23% decreases in the spheroid viability, respectively (Figure 5F). The platform can detect the toxicity of a low curcumin concentration (70 µM) after 35 h of incubation and a high curcumin concentration (500 µM) within a short amount of time (<7 h). However, this finding is incredibly difficult to discern through conventional colorimetric methods. In addition, the platform can be utilized for the highly sensitive detection of spheroid viability under short-term (63 h) and long-term (144 h) culture conditions in a real-time and non-destructive manner. With this platform, the integrity of the sample is not compromised. After 35 h of incubation, even the toxicity of a low curcumin concentration (70 μM) can be detected as a harmful effect. However, the spheroids are damaged in a short period of time (less than 7 h) upon exposure to a high curcumin concentration (500 μM). This damage is difficult to distinguish using traditional colorimetric methods because the HCGN platform is designed to support these methods. As a result, the HCGN platform has the potential to accelerate the identification of new medications for cancer. Therefore, it is a highly promising, label-free, and high-throughput drug screening method for 3D cell culture systems.

### 3.2. Stem Cell-Based Sensing and Monitoring for Differentiation

Stem cells are biological cells that can multiply through mitosis and develop into at least one type of specialized cell [123,124]. They exist in various types, including pluripotent stem cells (PSCs), mesenchymal stem cells (MSCs), and neural stem cells (NSCs), which can differentiate into specialized cells. They also possess various potentials for the applications of (i) utilization in different multicellular organisms, (ii) the potential to bridge a gap between the behaviors of cultivated cells and cells in vivo, and (iii) culture in an artificial environment and transformation into cell cultures with features compatible with their primary counterpart cells in various tissues. Stem cell differentiation is quite advantageous for obtaining specific cells of interest during drug development. These cells are then treated with drug candidates to verify the safety and efficacy of the drugs. However, these conventional approaches for controlling stem cell differentiation with soluble cues, such as growth factors, cytokines, and small biomolecules, are limited; consequently, the target specificity and efficiency of the differentiation are not yet successfully achieved. They also have critical limitations in terms of real-time and non-destructive monitoring of stem cell differentiation (e.g., organoid, osteogenesis, and neurogenesis). To address these concerns, many researchers have developed electrochemical cell-based biosensors for stem cell sensing and differentiation monitoring.

**Figure 6:**
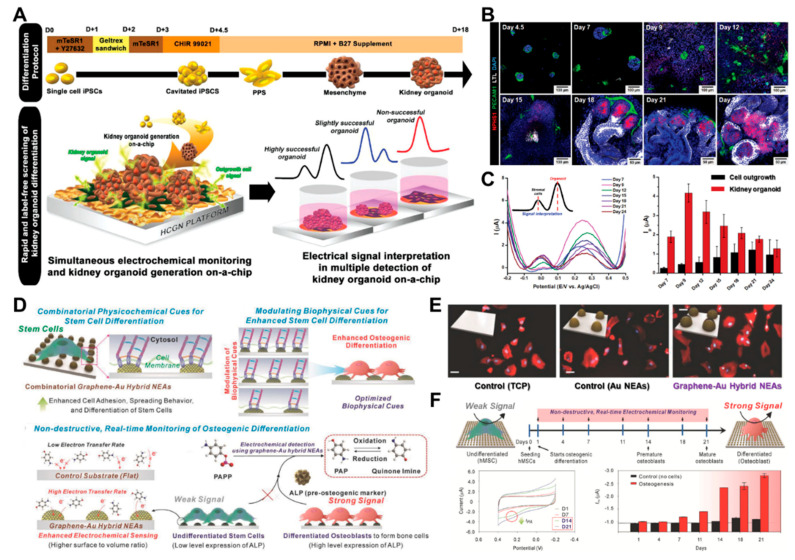
(**A**) Non-invasive monitoring of a kidney organoid on-a-chip using an electrochemical method for the detection of its successful and label-free differentiation. (**B**) Representative time-dependent images of NPHS1s (podocytes), LTLs (proximal tubules), and PECAM1s (vascular networks) during the kidney organoid differentiation. (**C**) Differential pulse voltammetry (DPV) graph of the kidney organoid differentiation from day 7 to day 24 (black bar, cell outgrowth; red bar, kidney organoid). (**D**) Multifunctional graphene–Au hybrid nanoelectrode arrays (NEAs) for the monitoring of osteogenic differentiation by enhancing electrochemical signals. (**E**) Representative images of human mesenchymal stem cells (hMSCs; scale bar, 50 µm). (**F**) Cyclic voltammetry (CV) graph of time-dependent monitoring of hMSCs during osteogenic differentiation. Reprinted with permission from [67]. Copyright 2022, Wiley Online Library; reprinted with permission from [114]. Copyright 2018, Wiley Online Library.

A human pluripotent stem cell (hPSC) is derived from somatic cells reprogrammed into an embryonic-like pluripotent state, thereby enabling the development of an unlimited source of any type of human cell needed for therapeutic purposes [125]. PSCs are widely used in therapeutics for disease modeling, regenerative medicine, and drug discovery [126]. Among the various applications, organoids, which are self-organized multicellular tissues derived in vitro from human PSCs, have recently emerged as intriguing models for human diseases. Thus, the differentiation should be monitored for detecting maturity without destroying the differentiated cells. PSCs are extremely sensitive to the ECM, which is a biological reservoir for cell growth and differentiation factors. Suhito et al. addressed this issue and proposed an HCGN platform modified on a transparent indium tin oxide (ITO) glass for the precise detection of hPSCs and the real-time monitoring of kidney organoid differentiation via simple electrochemical detection (e.g., differential pulse voltammetry, DPV; Figure 6A) [67]. This platform can detect PSC-specific electrical signals ranging from 21,000 cells to 157,000 cells; the limit of detection (LOD) of the proposed platform is 21,363 cells. Furthermore, the kidney organoid is successfully differentiated and assessed for maturation on this platform. In a previous study, immunostaining was performed with electrochemical detection up to day 24 in a 3-day time interval to assess the maturation of a kidney organoid and off-target cell generation (Figure 6B,C). On day 18, the major markers of podocytes (NPHS1) and proximal tubules (LTL) were strongly expressed. Interestingly, two peaks from this platform were detected at approximately 0 V (I_pO_) and 0.3 V (I_pK_) during the monitoring of the kidney organoid differentiation. This redox signal intensity trend was consistent with the expression levels of kidney-specific cell types, particularly CDH16, which is a well-known proximal tubule early transcription factor. Hence, in this proposed platform, I_pO_ and I_pK_ are used as indicators for off-target cell outgrowth and kidney organoid differentiation, respectively.

Given that the effects of the combinatorial physiochemical cues during stem cell differentiation are unknown, various materials (e.g., graphene, graphene oxide, and gold) are utilized to identify the biophysical cues and monitor the differentiation of stem cells into specific lineages [127]. To conquer these issues, Lee et al. proposed the multifunctional graphene–gold hybrid nanoelectrode arrays (graphene–Au NEAs) for the real-time monitoring of osteogenic differentiation derived from human mesenchymal stem cells (Figure 6D) **[117]**. The proposed platform is utilized in graphene–Au hybrid nanoelectrode arrays (NEAs) to monitor non-destructive real-time stem cell differentiations. Multifunctional graphene–Au hybrid NEAs are manufactured using laser interference lithography and physical vapor deposition techniques, followed by a surface treatment with a reduced graphene oxide. The presence of the reduced graphene oxide improves the cell adherence and spread in the absence of functionalization with ECM proteins, which can function as insulators and lessen the electrostatic repulsion (ET) between the electrodes and the electroactive molecules. Because of the superior biocompatibility and electrochemical performance of the graphene–Au hybrid NEAs, the osteogenic differentiation of human mesenchymal stem cells is effectively evaluated using an ALP-based enzymatic reaction, which is reported as osteogenic markers (Figure 6E). PAPP (p-aminophenyl phosphate) is added to the cells prior to the electrochemical monitoring. The ALP expressed on the cell hydrolyzes PAPP to generate electroactive p-aminophenol (PAP), and the redox reaction between PAP and quinone imine (QI) is detected using a cyclic voltammogram. The osteogenic development of human MSC is successfully observed in a non-destructive and real-time manner by using this technique (Figure 6F). Although stem cell therapy has emerged as a promising method in the field of biomedicine because of its unique ability to differentiate into multiple cell lineages, the need for destructive analysis processes, such as cell lysis and cell fixation, has considerably limited the development of additional clinical applications. This innovative electrochemical detection approach that uses graphene–Au hybrid NEAs can be a breakthrough in the preclinical studies of differentiated stem cells. Therefore, related research will greatly increase the number of stem cell differentiation tests by making practical, non-destructive monitoring tools available in real time.

## 4. Conclusions

This paper summarizes the relevant studies on electrochemical biosensors used to detect living cells (such as cancer and stem cells), determine anticancer drug efficacy, and monitor stem cell differentiation. These biosensors can be used for accurate, quick, and non-invasive measurements of various targets. Furthermore, several types of electrochemical biosensors (e.g., ECIS, FET, and potentiometric biosensors) are applied to detect living cells and target molecules. During the electrochemical measurements, these biosensors emit a measurable readout signal as a result of interactions between the living cells and the particular probes or composites. Therefore, the electrochemical approach is viewed as a platform for precise and rapid sensing before diagnostic and pharmaceutical developments. This approach can also be utilized to monitor live cells, including cancer cells (e.g., viability and anticancer drug development), and observe the status of stem cell differentiation based on their redox activities.

## 5. Future Perspectives

Electrical and electrochemical sensing systems show remarkable potential as simple, rapid, and economical tools to detect targets of interest. A representative example of such success is the glucose sensor, which enables the rapid, simple, and economical monitoring of glucose levels in the blood, thus, considerably benefiting patients with diabetes. These advantages of electrical biosensors will become more valuable for cell-based assays where characterizing living cells without harming their functions and viabilities is important. From the perspective of in vitro cell cultivation, the research trend has been changing from simple 2D cultures to complex 3D organ-mimicking constructs. This new type of culture platform includes stem cell-derived organoids (e.g., liver, heart, kidney, intestine, brain, and bone), patient-derived cancer spheroids, and organs-on-a-chip. Such three-dimensional tissue-like structures are hard to precisely characterize using conventional optical and colorimetric methods, including immunocytochemistry, flow cytometry, and optical imaging. Therefore, a new type of tool capable of non-invasive, non-destructive, and label-free analysis while preserving the distinct cell-to-cell and cell-to-ECM interactions should be developed. We hope that various electrical methods, including ECIS, FET, and potentiometric tools, will be excellent candidates or serve as a basis for designing new and efficient techniques that can specifically analyze next-generation in vitro organ-mimicking constructs.

## Figures and Tables

**Table 1:** Various types of electrochemical biosensors for live-cell detection.

Detection Method	Cell Line	Immobilisation Strategy	LOD (Target)	Ref
ECIS	SH-SY5Y, ND7/23 cells	PPy/s-MWCNTs/ AuNPs hydrogel/ITO	17 nM (Dopamine)	[78]
ECIS	MCF-7	Tb-MOF-on-Fe-MOF	58 μU/mL (CA125)	[79]
ECIS	HeLa	FA@UiO-66 nanocomposite/Au	90 cells/mL	[80]
ECIS	HeLa, MDCK, 293T	CdZnSeS QD/OMC	10.23 μM (H_2_O_2_)	[81]
ECIS	HeLa	Cu2O–CuO@GQDs	1 nM (Bisphenol A)	[82]
ECIS	LNCaP	BPene@PDA−SCX8	36 cells/mL	[83]
FET	PC12	PVC coated CNT-FET	1 nM (Potassium ion)	[84]
FET	U-251 MG	Graphene-based ISFET	1 μM (Potassium ion)	[85]
FET	HepG2	graphene foam FET	0.5 pM (ATP)	[86]
FET	HeLa	MoS_2_/RGO FET	1 pM (H_2_O_2_)	[87]
Amperometric	MCF-7	ZnMn_2_O_4_/rGO	0.012 μM (H_2_O_2_)	[88]
DPV	MCF-7	ITO/MWCNT/PDDAHA	5.94 pg/mL (CD44)	[89]
LSV	HepG2	GC/ZNBs/fMWCNT	35 nM (H_2_O_2_)	[90]
Amperometric	Patient-derived cancer	MnO_2_-NWs@Au-NPs/GF	1.9 μM (H_2_O_2_)	[91]

**Table 2:** Electrochemical biosensing platforms for anticancer drug testing.

Detection Method	Cell Lines	Immobilisation Strategy	Anticancer Drug	Ref
EIS	Cervical cancer (HeLa)	c-MWCNTs/AuNPs	Pinoresinol	[59]
Amperometric	Lung cancer (H1299)	HRP-AuNPs-MWNT	fMLP	[107]
CV	Liver cancer (HepG2)	G-quadruplex/hemin/Au /QZIF-67-2/GCE	Paclitaxel	[108]
SECM	Patient-derived cancer organoid (colorectal)	Fibrin-collagen gel	Bortezomib	[109]
SWV	Cervical cancer (HeLa)	Telomerase and dNTPs	Epigallocatechin gallate (EGCG)	[110]
Amperometric	Lung cancer (PC9)	PHF-MWNT-PB-Gox	Osimertini	[111]
EIS	Cervical cancer (HeLa)	C-Kemptide-modified AuNP /rGO-GCE	H-89	[112]

**Table 3:** Electrochemical biosensing platforms for stem cell detection.

Detection Method	Cell Lines	Differentiated Cells	LOD (Target)	Ref
DPV	Human embryonic stem cell	Undifferentiated stem cell	12,500 cells (ESC)	[57]
SWV	Human tissue	Cancer stem cell	0.5–1000 ng/mL (Oct4)	[113]
CV	Human mesenchymal stem cell	Osteogenic differentiation	0.03 unit/mL (ALP)	[114]
Amperometric	Human embryonic stem cell	Brain organoid	0.5 mM (glutamine)	[115]
EIS	Human neuroepithelial stem cell	Midbrain organoid	8 nM (epinephrine)	[116]
DPV	Mouse neural stem cell	Neuronal differentiation	134 nM (dopamine)	[117]
DPV	Bone mesenchymal stem cell	Cardiomyogenic differentiation	0.42 pg/mL (cTnI)	[118]

## Data Availability

Not applicable.
